# The type of lipid supplement has crucial implications for forage particle size in calf starter diets

**DOI:** 10.1186/s40104-023-00913-5

**Published:** 2023-09-04

**Authors:** Pedram Panahiha, Hamidreza Mirzaei-Alamouti, Mehdi Kazemi-Bonchenari, Mehdi Poorhamdollah, Mina Vazirigohar, Jörg R. Aschenbach

**Affiliations:** 1 Department of Animal Science, University of Zanjan, Zanjan, 45371-38791 Iran; 2 Department of Animal Science, Faculty of Agriculture and Natural Resources, Arak University, Arak, 38156-8-8349 Iran; 3 Department of Animal Science, College of Agriculture and Natural Resources, University of Tehran, Karaj, 31587-11167 Iran; 4 Zist Dam Group, University of Zanjan Incubator Center, Zanjan, 45371-38791 Iran; 5 Institute of Veterinary Physiology, Freie Universität Berlin, Oertzenweg 19B, 14163 Berlin, Germany

**Keywords:** Forage particle size, Lipid supplement, Oxidative stress, Ruminal functional development

## Abstract

**Background:**

Forage inclusion in starters of young dairy calves has become an acceptable strategy in the last decade. To compensate for the lower energy provided by forage, concurrent lipid supplementation can be proposed. However, ruminal microbial activity and forage digestibility may be decreased by lipid supplementation. We hypothesized that the composite effect of forage and lipid supplements may be dependent on forage particle size and the type of lipid supplement. Therefore, we evaluated the effect of long (LP; geometric mean, 4.97 mm) vs. short alfalfa hay particle sizes (SP; geometric mean, 1.26 mm) with either soybean oil (SBO) or palm fatty acids (PLF) as lipid source in a 2 × 2 factorial design with treatments SP-SBO, SP-PLF, LP-SBO, and LP-PLF. Treatments (*n* = 13 with 6 males and 7 females each) were offered to Holstein calves (3 days old) with equal amounts of lipid (25 g/kg DM) throughout the experimental period. The milk offering scheme (d 1 to 53) was equal for all groups. Data collection continued until 20 d post-weaning.

**Results:**

Interaction between forage particle size and lipid supplement was significant for the following readouts: the highest and lowest starter intakes during the pre-weaning period occurred in LP-PLF and LP-SBO, respectively. This was associated with similarly contrasting changes in average daily gain (ADG) during the post-weaning period, body weight at the end of experiment, withers height, digestibility of organic matter and neutral detergent fiber, and blood serum concentrations of glucose, beta-hydroxybutyrate, and insulin during the pre-weaning period. During both pre- and post-weaning periods, the highest and lowest urinary excretion of allantoin and total purine derivatives, representing microbial protein synthesis, were observed in LP-PLF and LP-SBO, respectively, indicating that those diets were most and least favorable for rumen development. Irrespective of forage particle size, supplemental SBO vs. PLF increased serum malondialdehyde as an oxidative stress indicator across periods, increased blood urea nitrogen and feed efficiency in the pre-weaning period, and reduced hip height during the post-weaning period.

**Conclusions:**

It can be concluded that feeding a rumen-inert, mostly saturated fatty acid source with alfalfa hay as long particle size is recommended with view on performance, whereas a combination soybean oil rich in unsaturated fatty acids should not be provided to milk-fed Holstein calves together with long particle forage. Feeding soybean oil and alfalfa hay as long particles is not advisable mainly due to lower starter consumption and impaired development of ruminal function. If dietary supplementation of soybean oil is applied, incorporation of forage as small particles should be preferred to support rumen development.

## Introduction

Forage inclusion in the starter feed of young dairy calves has been considered as a favorable feeding strategy in recent years. This feeding strategy aims to stabilize ruminal pH and to improve ruminal fermentation while also preventing hyperkeratinization and clumping of ruminal papillae [1]. In addition to ruminal effects, recent studies indicated that forage inclusion in starter feeds of young calves has potential to maintain proper metabolic and hormonal status with improvement of the somatotropic axis [2]. Contrary to these considerations, some worry that forage may displace concentrate intake and shift ruminal fermentation towards acetate rather than butyrate production, thus, delay ruminal papillae development in young ruminants [3]. Further arguments against starter provision with high forage content to young ruminants are expectations of reduced starter intake and body weight gain [4], reduced ruminal wall development and impaired nutrient transport and metabolism [5].

Favorable and unfavorable impacts of feeding forages in starter feed of calves on growth performance and rumen fermentation may be dependent on the source of forage, forage feeding level, method of processing, and its chemical composition [6, 7]. Furthermore, forage particle size (FPS) can also be an important issue for the response of young calves to forage inclusion in starters [7, 8]. As stated in NRC (2001), ruminants need dietary forage with an adequate particle size to enhance fiber digestion and maintain their health [9]. Although discussion is made extensively on forage intake in young dairy calves; however, no clear recommendation is available for FPS in the recommendations of the National Academies of Science, Engineering, and Medicine [10]. A shorter than optimum FPS may result in low dry-matter (DM) intake, decreased fiber digestibility, and impaired health status. The reciprocal with higher than recommended FPS may provoke feed sorting, excessive time spent for re-chewing, higher retention time of digesta and reduced fractional passage kinetics of particulates. Limited documents are available with respect to the effect of FPS on dairy calves’ performance.

Regardless of the particle size of forage incorporated in starter feeds, lower energy content of forage compared to concentrate can provide less energy in forage-based starter diets in comparison with all-concentrate starter feeds [4, 11]. The lower energy supplied through forage compared to concentrate may be considered as an obstacle in accelerated growth programs with early weaning in commercial dairy farms. Lipid supplementation can be applied as a strategy in forage-containing starters to compensate the energy deficit [12, 13]. In fact, it can be postulated that concurrent feeding of forage and lipid may be a practical way to support both stable ruminal conditions and high performance of young calves. The biological activities of lipids extend beyond serving as an energy source and maintaining normal biological functions; however, there is limited understanding of how they are utilized by developing dairy calves. In recent studies, oil supplementation to growing lambs’ diet improved ruminal health and development, feeding behavior and animal performance [14], as well as ruminal epithelial function and metabolism [15]. A better understanding of lipid metabolism and the impacts of supplementation of dietary fats or manipulations of energy sources may offer new ways to enhance development and improve performance of growing replacement heifers through nutritional strategies [16]. A negative interaction may be present between fiber and lipids in ruminants, which can be due to coating effects of lipids on fiber digestion and toxic effects on ruminal micro-organisms due to compounds produced by lipids metabolism [17]. The negative effect of lipids on fiber digestion may be more of a concern in dairy calves compared to mature ruminants due to their less developed microbial community. Some previous studies addressed the interaction effect of forage source and lipid supplement on performance of dairy calves [11, 13]; however, the combined effects of forage particle size and lipid supplement need to be evaluated in dairy calves. In addition, as stated in previous studies [12, 14, 16], lipid supplement may have different impacts from the oxidative stress perspective, which can eventually influence whole animal body metabolism. The interactive effect of FPS with different lipid supplement on the oxidative stress of young calves also needs to be evaluated in dairy calves. Therefore, the aim of the current study was to evaluate the interaction effect between different forage particle sizes (geometric mean particle lengths equal to 1.26 mm as SP vs. 4.97 mm as LP) and supplemental lipid source (SBO vs. PLF) on growth performance, blood metabolites, blood insulin concentration, oxidative stress indicators, and urinary purine derivatives in young dairy calves.

## Materials and methods

### Calves and management

The present study was conducted on a commercial dairy farm located in the Qazvin province, Iran. The experimental design, management protocols, and procedures were approved by the Animal Care Committee at the University of Zanjan, Iran (ID 1353). Fifty-two, healthy Holstein dairy calves (24 males and 28 females; 41.1 ± 2.9 kg BW at birth) were selected at 3 days of age and randomly allocated to 4 groups (13 calves per treatment; 6 males and 7 females, each) in a 2 × 2 factorial arrangement. Calves were housed in 1.3 m × 2.4 m individual pens. The floor of each pen was bedded with sand that was renewed every 24 h. Each calf was fed 5 L of colostrum during the first 12 h of life (2.5 L within 1.5 h after birth and 2.5 L in a second feeding) which was continued during the first 2 d of life. Colostrum quality was tested using a digital Brix refractometer (PAL-1, Atago Co., Ltd., Bellevue, WA, USA) and only colostrum with Brix scale greater than 22 was used. The experiment started on d 3. Calves were fed 4.5, 7.5, 2.5 and 1.5 L/d of whole milk from d 3 to 10, 11 to 40, 41 to 51 and 52 to 53, respectively. Fat, protein, lactose and total solid of milk samples were measured weekly by using an infrared spectrophotometer (FOSS Milk-O-Scan, FOSS Electric, Hillerod, Denmark). The average composition of consumed milk was 31.2 ± 0.7, 31.3 ± 0.3, 49.2 ± 0.3 and 118 ± 0.4 g/kg of fat, protein, lactose and total solid, respectively. All calves were weaned at 53 days of age but maintained on the experimental diets until 73 days of age.

### Experimental starter diets

Experimental diets were formulated according to NRC 2001 [9] recommendations to meet nutrient requirements, but differed in alfalfa hay particle sizes and lipid source according to treatment. Four experimental diets were: 1) small particles of alfalfa hay with soybean oil (SP-SBO); 2) small particles of alfalfa hay with palm fatty acids (SP-PLF); 3) long particles of alfalfa hay with soybean oil (LP-SBO); and 4) long particles of alfalfa hay with palm fatty acids (LP-PLF). The feed ingredients and the chemical composition of experimental starter diets are given in Table 1. A Penn State particle separator (Nasco, Fort Atkinson, WI, USA) equipped with 3 sieves (19, 8, and 1.18 mm) and a bottom pan were used to separate the differently chopped alfalfa hay for particle size analysis into long (> 19 mm), medium (8–19 mm), short (1.18–8 mm), and fine (< 1.18 mm) fractions [18]. The resulting geometric mean of particle size (GMPL) for the two alfalfa hay feed ingredients was calculated as described by the American Society of Agricultural Engineers [19]. These were 1.26 and 4.97 mm for chopped alfalfa hay with small and long particle sizes, respectively. The concentrate feed inclusive of lipid source was mixed well with forages and the diet was offered as total mixed ration throughout the study. The SBO was sourced from Naz Industrial (Vegetable Oil Co., Isfahan, Iran) and had the following fatty acid composition: C16:0, C18:0, C18:1, C18:2, C18:3, and other fatty acids equal to 12.1%, 5.2%, 21.8%, 51.2%, 8.1%, and 1.6%, respectively. The composition of PLF (rumen-inert; Energizer RP-10, IFFCO, Johor, Malaysia) was C12:0, C14:0, C16:0, C18:0, C18:1, and other fatty acids equal to 2.3%, 4.2%, 86.0%, 2.0%, 4.1%, and 1.4%, respectively. The starters were fed ad libitum from d 3 onwards in an amount that permitted at least 10% orts. The calves had free access to water throughout the experimental period.

**Table 1:** Experimental starter feed ingredients and chemical composition

Item	Treatments^1^			
	SP		LP	
	SBO	PLF	SBO	PLF
Ingredients, g/kg of DM				
Alfalfa hay (chopped, GMPL^2^: 1.26 mm)	100	100	0	0
Alfalfa hay (chopped, GMPL: 4.97 mm)	0	0	100	100
Barley grain, ground	140	140	140	140
Corn grain (coarsely ground)	420	420	420	420
Soybean meal	270	270	270	270
Soybean oil	25	0	25	0
Palm fatty acids	0	25	0	25
Calcium carbonate	8	8	8	8
Sodium bicarbonate	7	7	7	7
Di-calcium phosphate	6	6	6	6
Salt	4	4	4	4
Vitamin and mineral mix^3^	20	20	20	20
Chemical composition, g/kg of DM, unless stated otherwise				
Metabolizable energy^4^, Mcal/kg	2.94	2.94	2.94	2.94
CP	208	208	208	208
NDF	184	184	184	184
Ether extract (EE)	56.3	56.3	56.3	56.3
Non-fiber carbohydrate^5^	501	501	501	501
Ca	84	84	84	84
P	45	45	45	45

^1^Treatments: alfalfa hay provided as small particle size supplemented with soybean oil (SP-SBO); alfalfa hay provided as small particle size supplemented with palm fatty acids (SP-PLF); alfalfa hay provided as long particle size supplemented with soybean oil (LP-SBO); alfalfa hay provided as long particle size supplemented with palm fatty acids (LP-PLF)

^2^GMPL: Geometric mean of particle size

^3^Containing per kg: Vit A (IU) = 1,000,000, Vit D (IU) = 110,000, Vit E (IU) = 1,100 Ca (g) = 90, P (g) = 25, Mg (g) = 30, Zn (mg) = 1,700, Cu (mg) = 540, I (mg) = 110, Co (mg) = 80, Mn (mg) = 1,900, Se (mg) = 140

^4^Calculated according to NRC (2001) [9]

^5^Non-fibre carbohydrate was calculated as DM − (NDF + CP + EE + ash) (NRC, 2001) [9]

### Intake, feed efficiency, digestibility, and fecal score

Experimental diets were offered once daily at 0800 h and orts were collected and recorded the following morning (0730 h). The starter DM intake was calculated as the difference of offered starter feed and collected orts for each calf. The BW of calves was measured on d 3 (initial day of experiment) and then every 10 d during the study; BW recording was performed before feeding to avoid bias by gastrointestinal fill. Average daily gain (ADG) and feed efficiency (FE: kg of BW gain divided by kg of total DM intake; the latter being the sum of milk DM and starter DM) were calculated before (d 3–53) and after (d 54–73) weaning, and for the entire period (d 3–73). Samples of experimental diets and orts were dried at 60 °C for 48 h in a convection oven every 14 d. To determine chemical composition, subsamples of dried experimental starter diets and their orts were mixed thoroughly and ground in a mill (Ogaw Seiki CO., Ltd., Tokyo, Japan) to pass a 1-mm screen. Experimental diets and their orts were analyzed for DM (method No. 2001.12), ash (method No. 942.05), CP (method No. 991.20), and ether extract (EE; method No. 920.39) according to standard procedures [20]. To determine neutral detergent fiber (NDF), samples of experimental diets were analyzed by applying a heat-stable alpha-amylase without sodium sulfite addition and with correction for residual ash and N [21]. Apparent total tract digestibility of nutrients was measured during four congestive days at post-weaning period (d 69–72) using acid insoluble ash as an internal marker [22]. Fecal scoring was performed according to Ghorbani et al. [23] as 1 = firm, 2 = soft, 3 = soft and running, and 4 = watery.

### Skeletal growth parameters

Skeletal growth parameters of all calves, including withers height, body barrel, hip height, heart girth, and body length were measured at the beginning of experiment (d 3), at weaning (d 53) and at the last day of experiment (d 73) as described for young dairy calves [24].

### Blood metabolites, insulin, and oxidative stress indicators

Blood samples were obtained without preservative on d 35 (before weaning) and 71 (after weaning) from the jugular vein at 2 h after the morning meal and allowed to clot. Immediately after centrifugation of clotted samples at 3,000 × *g* for 15 min at 4 °C, serum was separated and stored at −20 °C until subsequent measurements. Serum concentrations of glucose, urea-N (BUN), triglycerides, cholesterol, and total protein were determined using autoanalyzer (UNICCO, 2100; Zistchemi Co., Tehran, Iran) with appropriate analytical kits (Pars-Azemun Co., Ltd., Karaj, Iran). Serum beta-hydroxybutyrate (BHB) was measured using a commercial kit (Abbott Diabetes Care Ltd.) and insulin concentration was measured using Insulin AccuBind ELISA (MonobindInc, Lake Forest, CA, USA) according to manufacturers' instructions.

Activities of glutathione peroxidase (GPx), superoxide dismutase (SOD), and concentration of malondialdehyde (MDA) in blood were measured as oxidative stress indicators using a plate reader (DANA 3200, Garny, Iran). Activity of GPx was evaluated by Paglia and Valentine's method [25] in whole blood, using RANSEL Kit (Randox, UK). In this kit, GPx catalyses the oxidation of glutathione cumene hydroperoxide. SOD activity was measured in haemolysate by an iodophenyl nitrophenol phenyltetrazolium chloride modified method (RANSOD Kit, Randox, UK). This method employs xanthine and xanthine oxidase to generate superoxide radicals which react with 2-(4-iodophenyl)-3-(4-nitrophenol)-5-phenyl-tetrazolium chloride to form a red formazan dye. SOD activity was then measured by the degree of inhibition of this reaction [26]. Measurement of MDA was performed by the thiobarbituric acid method, for which reactive substances were measured with thiobarbituric acid as peroxidation index. The basis of this measurement is the MDA reaction with thiobarbituric acid, which results in the formation of pink colour, and is calculated by measuring the intensity of the color produced at 520 nm [27].

### Urinary purine derivatives and microbial protein synthesis

For estimating microbial protein synthesis (MPS) in the rumen, the spot sampling technique was used, which is based on purine derivatives (PD) excretion in urine. Total urine volume was estimated as BW × 26.8/creatinine concentration (mg/L) in dairy calves less than 4 months of age, as recently reported by Dennis et al. [28]. Urine spot samples (approximately 10 mL) were collected at four consecutive days during the pre-weaning period (41–44 d) and post-weaning period (66–69 d) between 0900 and 1100 h and 1500 and 1700 h at the time that calves had spontaneous urination. A sub-sample of 5 mL of each sample was diluted immediately with 45 mL of 0.036 N sulfuric acid and stored at –20 °C for analysis. Sub-samples were pooled per animal and sampling period before analysis. The analysis was performed as previously reported [29]. In brief, the concentrations of creatinine (Kit No. 555-A; Sigma Chem. Co., St. Louis, MO, USA) and uric acid (Kit No. 685–50 Sigma Chem. Co.) were measured using spectrophotometer (UV-2600i, Shimadzu, Japan) in accordance with the manufacturer's instructions. For allantoin determination the high-performance liquid chromatography as model 10 A high-performance liquid chromatograph (Shimadzu, Kyoto, Japan) equipped with a UV spectrophotometric detector set at 220 nm was used [30]. The daily endogenous PD was considered to be 705 µmol/kg of BW^0.75^ according to previous suggestions in dairy calves [31]. To prevent overestimation of MPS due to PD contained in the consumed milk, 280 μmol PD/L of milk were subtracted from the total PD during the pre-weaning period [32]. For post-weaning calves, no correction was made, and it was assumed that the total PD via urine minus endogenous PD was of microbial origin, as reported in a previous study of post-weaning calves [30]. The MPS in the rumen of calves was then calculated from daily urinary PD output using the following equation described by Chen and Gomes [33]: Microbial N (g N/d) = *X* (mmol/d) × 70/(0.116 × 0.83 × 1,000), where *X* is the microbial purine uptake (mmol/d), 70 is the N content of the purine coefficient (mg N/mmol), 0.116 is the ratio of purine N to total N in mixed rumen microbiota (which is 11.6:100), and 0.83 is the average digestibility of microbial purines [33].

### Statistical analysis

All data were analyzed using PROC MIXED of SAS (version 9.1; SAS Institute, Cary, NC, USA). Individual calf was used as experimental unit. When repeated measurements were available within a period (starter DM intake, ADG, and FE), data were additionally treated as repeated measures per period. Data at each time point/period were tested using the following model: ***Y***_*ijk*_ = ***μ*** + ***FPS***_*i*_ + ***Fat***_*j*_ + *[****FPS*** × ***Fat****]*_*ij*_ + *β [****X***_***i***_*—*$\overline{\boldsymbol X}$*]* + *ε*_*ijk*_ where ***Y***_*ijk*_ is the dependent variable; ***µ*** is the overall mean; ***FPS***_*i*_ is the effect of alfalfa hay particle size (small vs. long particle sizes); ***Fat***_*j*_ is the effect of lipid source (SBO vs. PLF; and *[****FPS*** × ***Fat****]*_*jk*_ is the two-way interaction effect of alfalfa hay particle size and lipid source; *β [****X***_*i*_*—*$\overline{\boldsymbol X}$*]* is considered as covariate and *ε*_*ijk*_ is the overall error term. When data were available at different time points/periods (all data except milk intake, total dry matter intake and nutrient digestibility), the effects across time points/periods were also tested in a second step using the model ***Y***_*ijkl*_ = ***μ*** + ***FPS***_*i*_ + ***Fat***_*j*_ + *[****FPS*** × ***Fat****]*_*ij*_ + ***T***_*k*_ + *[****FPS*** × ***T****]*_*ik*_ + *[****Fat*** × ***T****]*_*jk*_ + *[****FPS*** × ***Fat*** × ***T****]*_*ijk*_ + *β [****X***_***i***_*—*$\overline{\boldsymbol X}$*]* + *ε*_*ijkl*_ where ***T***_*k*_ is the effect of time (pre-weaning vs. post-weaning period or the three time points of BW measurement); and *[****FPS*** × ***T****]*_*ij*_, *[****Fat*** × ***T****]*_*ik*_, and *[****FPS*** × ***Fat*** × ***T****]*_*ijk*_ are the two- and three-way interaction effects of time with the factors alfalfa hay particle size and lipid source. The effects of sex were also tested, including all two- and three-way interactions with the factor sex. Because these were not significant for any of the variables tested, except for a tendency regarding withers height (*P* = 0.09), sex was completely excluded from the model. An autoregressive (order 1) covariance structure was chosen according to the Akaike and Bayesian information criteria. Data were analyzed for three periods including before weaning (d 3–53), after weaning (d 54–73) and the entire period (d 3–73). The initial measurements (d 3) were included as a covariate for the analysis of BW and skeletal growth parameters at weaning and at the end of experiment. The fecal score was square-root transformed for better homogeneity of the distribution of residuals. Tukey’s multiple range tests were used to determine the differences among experimental diets means. Results were considered to be significant when *P* ≤ 0.05, and a tendency was considered when 0.05 < *P* ≤ 0.10. All data are reported as least square means with pooled standard error of mean (SEM).

## Results

### Stater intake, gain and efficiency, fecal score, and nutrient digestibility

During the pre-weaning period, starter intake was lower for SBO compared to PLF (*P* = 0.001) and, additionally, an interaction was found for FPS × lipid supplement with the highest starter intake found in LP-PLF and lowest starter intake found in LP-SBO (*P* = 0.048; Table 2). No effect of FPS, lipid supplement, or their interaction was detected during the post-weaning period. Milk intake did not differ among experimental treatments; however, because of lower starter intake in calves fed SBO diets, TDMI (milk DM + starter DM) was lower in these calves compared to calves receiving PLF diets (*P* = 0.001). Post-weaning and over the entire period, ADG was affected by lipid supplement (*P* = 0.012 and *P* = 0.008, respectively) with an interaction between FPS × lipid supplement (*P* = 0.037 and *P* = 0.026, respectively), resulting in highest ADG in LP-PLF and lowest ADG in LP-SBO. In accordance with the changes for ADG, BW at weaning and final BW were affected by lipid supplement (*P* = 0.003 and *P* = 0.001, respectively) with an interaction between FPS × lipid supplement over the entire period (*P* = 0.014), leading to highest final BW in LP-PLF and lowest final BW in LP-SBO. Feed efficiency was lower during the pre-weaning period (*P* = 0.048) and entire period of experiment (*P* = 0.029) in calves that received SBO compared to PLF.

**Table 2:** Least square means for starter intake, average daily gain, feed efficiency, and nutrient digestibility in dairy calves fed different alfalfa particle sizes (small particles vs. long particles) and lipid supplements (soybean oil and palm fatty acids) (*n* = 13 calves per treatment)

Item	Treatments^1^				SEM	*P*-value^2^						
	SP		LP			FPS	Lipid	FPS × Lipid	T	FPS × T	Lipid × T	FPS × Lipid × T
	SBO	PLF	SBO	PLF								
Starter feed intake, g/d												
Pre-weaning (d 3–53)	435^b^^,BC^	573^ab,BC^	336^c^^,C^	606^a^^,B^	34.7	0.33	0.001	0.048				
Post-weaning (d 54–73)	2,265^A^	2,259^A^	2,307^A^	2,438^A^	90.1	0.14	0.33	0.31				
Entire period (d 3–73)	958	1,056	899	1,142	97.2	0.88	0.72	0.44	0.003	0.006	0.002	0.007
Milk intake, g/d	676	673	671	675	23.4	0.99	0.98	0.97				
Total dry matter intake (milk + starter), g/d	1,112	1,249	1,011	1,281	41.3	0.41	0.001	0.11				
Average daily gain, g/d												
Pre-weaning (d 3–53)	451^C^	540^BC^	436^C^	624^B^	34.2	0.25	0.004	0.093				
Post-weaning (d 54–73)	728^b^^,AB^	778^b^^,AB^	658^c^^,B^	839^a^^,A^	38.8	0.89	0.012	0.037				
Entire period (d 3–73)	530^b^	608^b^	499^c^	685^a^	29.6	0.37	0.008	0.026	0.001	0.003	0.002	0.002
Body weight												
Initial (d 3)	41.1^F^	40.9^F^	41.7^F^	41.2^F^	1.30	0.66	0.75	0.91				
Weaning (d 53)	63.2^E^	67.4^E^	63.5^E^	72.4^DE^	1.81	0.12	0.003	0.072				
Final (d 73)	78.1^b,BC^	82.9^b,B^	76.2^c,CD^	89.2^a,A^	1.96	0.14	0.001	0.014	0.004	0.009	0.005	0.003
Feed efficiency^3^												
Pre-weaning (d 3–53)	0.402^AB^	0.431^AB^	0.429^AB^	0.486^A^	0.0357	0.10	0.047	0.51				
Post-weaning (d 54–73)	0.338^BC^	0.354^B^	0.302^C^	0.339^BC^	0.0413	0.22	0.202	0.62				
Entire period (d 3–73)	0.384	0.409	0.393	0.445	0.0449	0.21	0.029	0.45	0.006	0.046	0.001	0.001
Fecal score												
Pre-weaning (d 3–53)	2.0^A^	1.6^B^	1.8^AB^	1.6^AB^	0.54	0.13	0.001	0.43				
Post-weaning (d 54–73)	1.3^C^	1.3^C^	1.4^BC^	1.4^BC^	0.39	0.47	0.97	0.85				
Entire period (d 3–73)	1.9	1.5	1.8	1.5	0.48	0.32	0.004	0.45	0.001	0.081	0.023	0.008
Nutrient digestibility, %												
Organic matter	70.4^b^	71.3^ab^	66.8^c^	75.9^a^	1.83	0.72	0.026	0.006				
Ether extract	84.6	86.0	85.5	86.1	1.31	0.71	0.38	0.66				
Neutral detergent fiber	42.4^ab^	39.1^b^	32.5^c^	49.9^a^	1.84	0.90	0.034	0.042				
Crude protein	56.6	62.9	68.5	65.3	1.36	0.46	0.20	0.97				

^1^Treatments: alfalfa hay provided as small particle size supplemented with soybean oil (SP-SBO); alfalfa hay provided as small particle size supplemented with palm fatty acids (SP-PLF); alfalfa hay provided as long particle size supplemented with soybean oil (LP-SBO); alfalfa hay provided as long particle size supplemented with palm fatty acids (LP-PLF)

^2^Statistical comparisons: Forage particle size (FPS) = small versus long particle sizes; Lipid = soybean oil versus palm fatty acids; FPS × Lipid = interaction between alfalfa hay particle sizes and supplemental lipid; T = time; FPS × T = forage particle size × time; Lipid × T = lipid source × time; FBS × Lipid × T = forage particle size × lipid source × time

^3^kg of body weight/kg of total dry matter intake

^a−c^Different small letters indicate differences of a variable within one period (interaction FPS × Lipid; *P* ≤ 0.05)

^A−F^Different capital letters indicate differences of a variable across periods (interaction FPS × Lipid × T; *P* ≤ 0.05)

Fecal score was higher (i.e., calves had looser feces) when calves were fed SBO in comparison to PLF during pre-weaning (*P* = 0.001) and the entire experiment (*P* = 0.004; Table 2).

Regarding the time effect, starter feed intake, TDMI, ADG, and BW all increased from the pre-weaning to the post-weaning period (*P* = 0.003, 0.009, 0.001, and 0.004, respectively) with interactions for FPS × lipid × time (*P* = 0.007, 0.005, 0.002, and 0.003, respectively) that largely reflected the interactions observed in individual periods. Feed efficiency and fecal scores decreased from pre-weaning to post-weaning (*P* = 0.006 and 0.001, respectively) with an FPS × lipid × time interaction (*P* = 0.001 and 0.008, respectively) indicating that despite a missing two-way interaction of FPS × lipid, these factors separated differently when additionally considering the growing stage of the animal. For feed efficiency, the highest and lowest efficiencies were observed in LP-PLF pre-weaning and LP-SBO post-weaning, respectively. For fecal score, the highest value was observed in SP-SBO pre-weaning; however, this value improved to a commonly low level observed across treatments in the post-weaning period.

Digestibility of ether extract was not influenced by FPS. However, the digestibility of OM (*P* = 0.026) and NDF (*P* = 0.034) were lower in calves fed SBO compared to calves receiving PLF with an interaction showing that digestibility of OM (*P* = 0.006) and NDF (*P* = 0.042) was highest in LP-PLF and lowest in LP-SBO (Table 2).

### Structural growth indices

Withers height at weaning and at the end of experiment were greatest in LP-PLF and lowest in LP-SBO as supported by FPS × lipid supplement interactions (*P* = 0.001 and 0.003, respectively; Table 3). Body barrel was greater in calves fed long particles compared to calves fed short particles of alfalfa hay (post-weaning; *P* = 0.039). Feeding SBO to calves resulted in a tendency for lower hip height at weaning (*P* = 0.075) and lower hip height at the final day of experiment (*P* = 0.028). No effect of FPS, lipid supplement, or their interaction was found for body length, heart girth, and hip height.

**Table 3:** Least square means for structural growth indicators (cm) in dairy calves fed different alfalfa particle size (small particles vs. long particles) and lipid sources (soybean oil and palm fat) (*n* = 13 calves per treatment)

Item	Treatments^1^				SEM	*P*-value^2^						
	SP		LP			FPS	Lipid	FPS × Lipid	T	FPS × T	Lipid × T	FPS × Lipid × T
	SBO	PLF	SBO	PLF								
Body barrel												
d 53	112	113	111	112	5.4	0.44	0.78	0.74				
d 73	114	115	118	119	6.1	0.039	0.19	0.55				
Overall	113	114	114	115	5.8	0.048	0.67	0.71	0.007	0.54	0.093	0.69
Body length												
d 53	60.0	59.1	59.8	60.2	0.84	0.99	0.98	0.82				
d 73	62.8	63.3	64.2	63.8	0.91	0.55	0.91	0.66				
Overall	61.7	61.1	62.2	61.8	0.86	0.50	0.62	0.89	0.003	0.74	0.77	0.44
Heart girth												
d 53	98.6	97.2	97.5	99.2	1.19	0.62	0.91	0.14				
d 73	105	104	103	107	1.41	0.95	0.26	0.31				
Overall	101	101	100	102	1.50	0.78	0.34	0.064	0.001	0.71	0.42	0.94
Withers height												
d 53	94.9^ab^	92.2^b^	90.7^c^	96.8^a^	1.52	0.84	0.083	0.001				
d 73	99.0^ab^	96.6^b^	96.1^b^	100^a^	1.93	0.47	0.37	0.003				
Overall	97.0^b^	94.4^bc^	93.6^c^	98.7^a^	1.85	0.52	0.05	0.018	0.009	0.73	0.48	0.34
Hip height												
d 53	90.6	91.2	90.9	93.0	1.13	0.12	0.075	0.22				
d 73	94.6	96.5	95.8	96.3	1.28	0.33	0.028	0.21				
Overall	92.7	93.8	93.3	94.6	1.30	0.052	0.019	0.85	0.004	0.48	1.00	0.073
Hip width												
d 53	19.0	18.3	18.4	18.6	0.78	0.83	0.63	0.44				
d 73	20.1	19.8	20.7	20.3	0.80	0.30	0.51	0.79				
Overall	19.5	19.0	19.6	19.4	0.73	0.53	0.41	0.73	0.001	0.36	0.88	0.47

^1^Treatments: alfalfa hay provided as small particle size supplemented with soybean oil (SP-SBO); alfalfa hayprovided as small particle size supplemented with palm fat (SP-PLF); alfalfa hay provided as long particle sizesupplemented with soybean oil (LP-SBO); alfalfa hay provided as long particle size supplemented with palm fat (LP-PLF)

^2^Statistical comparisons: Forage particle size (FPS) = small versus long particle sizes; Lipid = soybean oil versus palm fatty acids; FPS × Lipid = interaction between alfalfa hay particle sizes and supplemental lipid; T = time; FPS × T= forage particlesize × time; Lipid × T= lipid source × time; FBS × Lipid × T = forage particle size × lipid source × time

^a–c^Different small letters indicate differences of a variable within one period (interaction FPS × Lipid; *P* ≤ 0.05)

As expectable from the accelerated BW gain in the post-weaning period described in the previous sub-section, all structural growth indices increased from the pre-weaning to the post-weaning period (all *P* ≤ 0.01); however, without any two- or three-way interactions for the factor time.

### Blood metabolites, insulin, and oxidative stress indicators

The blood serum concentrations of glucose (*P* = 0.029) and BHB (*P* = 0.077) were or tended to be affected by lipid supplement during the pre-weaning period with FPS × lipid supplement interactions (*P* = 0.003 and 0.009, respectively), showing that their concentrations were highest in LP-PLF and lowest in LP-SBO. During the post-weaning period, the serum concentration of glucose tended to be lower (*P* = 0.056) and the concentration of BHB were lower (*P* = 0.028) in calves receiving SBO (Table 4). Blood urea nitrogen was lower in calves fed PLF compared to SBO during the pre-weaning period (*P* = 0.035). Other blood chemical items such as cholesterol, total protein, and triglycerides were not affected by FPS, lipid supplement or their interaction.

**Table 4:** Least square means for blood metabolites and insulin concentration in dairy calves fed starters containing alfalfa hay with different particle sizes (small vs. long particles) with different lipid supplements (SBO vs. PLF, both 2.5% DM basis) (*n* = 13 calves per treatment)

Item	Treatments^1^				SEM	*P*-value^2^						
	SP		LP			FPS	Lipid	FPS × Lipid	T	FPS × T	Lipid × T	FPS × Lipid × T
	SBO	PLF	SBO	PLF								
Glucose, mg/dL												
d 53	97.8^ab,B^	94.2^b,B^	89.9B^c,C^	109.0^aA^	5.09	0.26	0.029	0.003				
d 73	64.1^D^	72.9^CD^	69.2^CD^	76.6^C^	3.53	0.25	0.056	0.88				
Overall	81.0^ab^	83.4^b^	79.5^c^	93.7^a^	4.94	0.104	0.004	0.036	0.008	0.98	0.90	0.024
BHB, mmol/L												
d 53	0.12^b,CD^	0.11^b,CD^	0.08^c,D^	0.15^a,C^	0.03	0.908	0.077	0.009				
d 73	0.25^B^	0.33^A^	0.31^AB^	0.35^A^	0.05	0.157	0.028	0.466				
Overall	0.19	0.22	0.19	0.25	0.04	0.188	0.017	0.495	0.003	0.22	0.26	0.052
Total protein, g/dL												
d 53	7.22	7.16	7.12	7.28	0.057	0.970	0.818	0.606				
d 73	6.96	7.11	6.61	6.56	0.049	0.127	0.814	0.695				
Overall	7.08	7.14	6.86	6.92	0.061	0.195	0.736	0.985	0.036	0.18	0.92	0.51
Triglyceride, mg/dL												
d 53	56.1	43.0	60.1	56.3	1.98	0.14	0.14	0.43				
d 73	33.5	26.3	28.0	30.2	0.79	0.83	0.51	0.21				
Overall	44.8	34.7	44.0	43.1	1.07	0.25	0.10	0.17	0.006	0.18	0.35	0.96
Cholesterol, mg/dL												
d 53	103	107	109	116	6.5	0.29	0.43	0.86				
d 73	74.6	70.1	80.2	76.2	4.23	0.35	0.51	0.64				
Overall	72.3	105	78.1	112	5.98	0.15	0.91	0.87	0.001	0.93	0.30	0.94
Blood urea nitrogen, mg/dl												
d 53	24.2	22.9	24.7	21.8	1.34	0.69	0.035	0.43				
d 73	16.5	18.2	17.8	16.8	1.02	0.94	0.88	0.27				
Overall	20.4	20.6	21.3	19.4	1.17	0.87	0.23	0.18	0.002	0.87	0.13	0.69
Insulin, IU/L												
d 53	8.16^b,B^	7.41^b,BC^	6.63^c,C^	9.65^a,AB^	0.614	0.357	0.006	0.001				
d 73	9.96^AB^	11.1^A^	10.2^A^	10.9^A^	0.900	0.910	0.038	0.79				
Overall	9.0^b^	9.26^b^	8.36^c^	10.3^a^	0.893	0.554	0.012	0.001	0.007	0.55	0.88	0.009

^1^Treatments: alfalfa hay provided as small particle size supplemented with soybean oil (SP-SBO); alfalfa hay provided as small particle size supplemented with palm fatty acids (SP-PLF); alfalfa hay provided as long particle size supplemented with soybean oil (LP-SBO); alfalfa hay provided as long particle size supplemented with palm fatty acids (LP-PLF)

^2^Statistical comparisons: Forage particle size (FPS) = small versus long particle sizes; Lipid = soybean oil versus palm fatty acids; FPS × Lipid = interaction between alfalfa hay particle sizes and supplemental lipid; T = time; FPS × T = forage particle size × time; Lipid × T = lipid source × time; FBS × Lipid × T = forage particle size × lipid source × time

^a−c^Different small letters indicate differences of a variable within one period (interaction FPS × Lipid; *P* ≤ 0.05)

^A−D^Different capital letters indicate differences of a variable across periods (interaction FPS × Lipid × T; *P* ≤ 0.05)

The blood serum insulin concentration was affected by lipid supplement (*P* = 0.006) and an interaction of FPS × lipid supplement during the pre-weaning period (*P* = 0.001; Table 4) with lowest concentrations found for LP-SBO and highest concentrations found for LP-PLF. In the post-weaning period, serum insulin concentration was reduced by supplemental SBO compared to PLF (*P* = 0.038).

When further tested for the factor time, the concentrations of glucose total protein, triglycerides, cholesterol, and blood urea nitrogen decreased (*P* = 0.008, 0.036, 0.006, 0.001, and 0.002 respectively) whereas the concentrations of BHB and insulin increased (*P* = 0.003 and 0.007, respectively) from pre-weaning to post-weaning. Three-way interactions of FPS × lipid × time existed for glucose, BHB and insulin (*P* = 0.024, 0.052 and 0.009, respectively), which largely reflected the interaction of FPS × lipid observed in the pre-weaning period.

Oxidative stress indicators GPx and SOD did not differ among experimental treatments; however, MDA was higher in calves receiving SBO compared to PLF during the pre- (*P* = 0.024) and post-weaning periods (*P* = 0.005) (Table 5). Malondialdehyde, GPx and SOD all increased from the pre-weaning to the post-weaning period (*P* = 0.007, 0.034 and 0.052, respectively) without any two-or three-way interactions.

**Table 5:** Least square means for oxidative stress indicators in whole blood of dairy calves fed starters containing alfalfa hay with different particle sizes (small vs. long particles) with different lipid supplements (SBO vs. PLF, both 2.5% DM basis) (*n* = 13 calves per treatment)

Item	Treatments^1^				SEM	*P*-value^2^						
	SP		LP			FPS	Lipid	FPS × Lipid	T	FPS × T	Lipid × T	FPS × Lipid × T
	SBO	PLF	SBO	PLF								
Glutathione peroxidase, IU/g Hb												
d 53	250	247	251	242	4.8	0.60	0.21	0.58				
d 73	297	289	299	293	5.1	0.47	0.14	0.88				
Overall	278	261	270	269	5.0	0.36	0.19	0.56	0.034	0.57	0.21	0.19
Superoxide dismutase, IU/g Hb												
d 53	2,267	2,351	2,240	2,215	83.8	0.34	0.73	0.52				
d 73	2,435	2,429	2,414	2,438	92.2	0.78	0.80	0.75				
Overall	2,390	2,389	2,374	2,364	91.6	0.54	0.70	0.34	0.052	0.60	0.43	0.27
Malondialdehyde, µmol/L												
d 53	2.41	1.81	2.16	1.89	0.093	0.11	0.024	0.39				
d 73	4.78	4.13	4.81	3.96	0.144	0.347	0.005	0.22				
Overall	3.89	2.90	3.07	2.17	0.132	0.236	0.062	0.78	0.007	0.79	0.093	0.45

^1^Treatments: alfalfa hay provided as small particle size supplemented with soybean oil (SP-SBO); alfalfa hay provided as small particle size supplemented with palm fatty acids (SP-PLF); alfalfa hay provided as long particle size supplemented with soybean oil (LP-SBO); alfalfa hay provided as long particle size supplemented with palm fatty acids (LP-PLF)

^2^Statistical comparisons: Forage particle size (FPS) = small versus long particle sizes; Lipid = soybean oil versus palm fatty acids; FPS × Lipid = interaction between alfalfa hay particle sizes and supplemental lipid; T = time; FPS × T = forage particle size × time; Lipid × T = lipid source × time; FBS × Lipid × T = forage particle size × lipid source × time

### Purine derivatives excretion and MPS

Urine volume and urinary creatinine excretion were not affected by FPS, lipid supplement, and their interaction (Table 6). However, urinary excretion of allantoin, total PD and, hence, MPS were affected by lipid supplement during the pre-weaning (*P* = 0.021, *P* = 0.036, and *P* = 0.036, respectively) and post-weaning periods (*P* = 0.004, *P* = 0.003, and *P* = 0.003, respectively). Moreover, interactions between FPS × lipid supplement showed that urinary allantoin, PD, and MPS were lowest for LP-SBO and highest for LP-PLF during the pre-weaning (*P* = 0.004, *P* = 0.008 and *P* = 0.008, respectively) and post-weaning periods (*P* = 0.031, *P* = 0.042, and *P* = 0.042, respectively). Creatinine, urine volume, allantoin, uric acid, total PD, and MPS all increased from the pre-weaning to the post-weaning period (*P* = 0.004, 0.001, 0.009, 0.007, 0.005, respectively). Of note, lipid × time interactions for allantoin, total PD and MPS (*P* = 0.008, 0.001, and 0.001, respectively) indicated that their values increased most prominently in the PLF groups from pre-weaning to post-weaning.

**Table 6:** Least square means for urinary purine derivatives in dairy calves fed starters containing alfalfa hay with different particle sizes (small vs. long particles) with different lipid supplements (SBO vs. PLF, both 2.5% DM basis) (*n* = 13 calves per treatment)

Item	Treatments^1^				SEM	*P*-value^2^						
	SP		LP			FPS	Lipid	FPS × Lipid	T	FPS × T	Lipid × T	FPS × Lipid × T
	SBO	PLF	SBO	PLF								
Creatinine, mg/dL												
d 41–44	120	118	111	114	8.5	0.13	0.85	0.65				
d 66–69	138	133	139	143	12.7	0.26	0.94	0.26				
Overall	129	126	124	128	11.0	0.73	0.84	0.26	0.004	0.068	0.93	0.64
Urine volume, L/d												
d 41–44	1.21	1.25	1.31	1.27	0.079	0.289	0.953	0.470				
d 66–69	1.71	1.78	1.71	1.64	0.146	0.304	0.891	0.229				
Overall	1.45	1.52	1.51	1.47	0.106	0.945	0.951	0.167	0.001	0.13	0.88	0.65
Allantoin, mmol/d												
d 41–44	7.79^ab^	7.56^ab^	6.24^c^	8.28^a^	0.694	0.289	0.021	0.004				
d 66–69	13.1^b^	15.8^ab^	12.1^c^	17.7^a^	1.58	0.52	0.004	0.031				
Overall	10.4^b^	11.7^ab^	9.2^c^	13.0^a^	1.70	0.99	0.007	0.003	0.009	0.27	0.008	0.69
Uric acid, mmol/d												
d 41–44	0.73	0.76	0.88	0.79	0.022	0.101	0.454	0.462				
d 66–69	1.16	1.19	1.25	1.18	0.082	0.622	0.608	0.385				
Overall	0.95	0.94	1.06	0.98	0.079	0.110	0.373	0.256	0.007	0.43	0.96	0.79
Total PD, mmol/d												
d 41–44	8.53^ab^	8.28^ab^	7.11^c^	9.08^a^	0.894	0.436	0.036	0.008				
d 66–69	14.3^b^	17.0^ab^	13.4^c^	18.9^a^	1.73	0.51	0.003	0.042				
Overall	11.4^b^	12.6^ab^	10.2^c^	13.9^a^	1.59	0.86	0.014	0.028	0.005	0.33	0.001	0.72
MPS, g/d^3^												
d 41–44	45.6^ab^	44.3^ab^	38.0^c^	48.5^a^	2.64	0.43	0.036	0.008				
d 66–69	76.8^b^	91.2^ab^	70.4^c^	101^a^	6.78	0.51	0.003	0.042				
Overall	61.2^b^	67.8^ab^	54.9^c^	74.8^a^	5.90	0.86	0.010	0.023	0.005	0.33	0.001	0.72

^1^Treatments: alfalfa hay provided as small particle size supplemented with soybean oil (SP-SBO); alfalfa hay provided as small particle size supplemented with palm fatty acids (SP-PLF); alfalfa hay provided as long particle size supplemented with soybean oil (LP-SBO); alfalfa hay provided as long particle size supplemented with palm fatty acids (LP-PLF)

^2^Statistical comparisons: Forage particle size (FPS) = small versus long particle sizes; Lipid = soybean oil versus palm fatty acids; FPS × Lipid = interaction between alfalfa hay particle sizes and supplemental lipid; T = time; FPS × T = forage particle size × time; Lipid × T = lipid source × time; FBS × Lipid × T = forage particle size × lipid source × time

^3^Microbial protein synthesis (MPS) was estimated from urinary purine derivatives (PD) excretion based on Chen and Gomes [33] as MPS (g/d) = 70 × PD (mmol/d)/(0.85 × 0.116 × 0.83 × 1,000) × 6.25

^a−c^Different small letters indicate differences of a variable within one period (interaction FPS × Lipid; *P* ≤ 0.05)

## Discussion

The current research evaluated the effects of forage particle sizes (long particles vs. small particles) in combination with two lipid supplements (SBO vs. PLF) in starter diets of young dairy calves on starter intake, oxidative stress indicators, microbial protein synthesis, and growth performance. The experiment covered the pre-weaning and post-weaning periods. These periods greatly differ as weaned calves have a better-developed rumen and higher starter intake [8, 24] with the consequence that blood metabolites are more similar to a mature ruminant [30]. This was reflected by higher BHB and insulin concentrations and lower blood glucose concentrations compared to pre-weaned calves. Due to the higher feed intakes, calves post weaning also had accelerated growth compared to pre-weaned calves. Importantly, the joined consideration of these periods provided a coherent picture of the interaction effects of lipid source and FPS. Francisco et al. [34] had evaluated such effects previously in lambs; however, limited work is published on this interaction in dairy calves.

We chose a forage inclusion level (on a DM basis) of 10% in accordance with Aragona et al. [35] who suggested that 5% forage incorporation constitutes the minimum requirement to support optimum development of ruminal fermentation. With respect to FPS, previous studies reported beneficial effects of increasing the particle size of alfalfa hay on calf performance and nonnutritive oral behaviors [36] as well as starter intake and weaning weight (DM basis) [8]. Omidi-Mirzaei et al. [37] evaluated wheat straw particle sizes when calves were fed texturized starters and also reported improved feeding behavior and ruminal fermentation when the content of fine particles was reduced. The novel finding in the current study is that lipid supplement becomes decisive for the effect of FPS when diets are lipid-supplemented. A positive effect of long FPS could only be observed when a rumen-inert source of fatty acids (PLF) was used for dietary fat supplementation. For example, animals in the LP-PLF group had higher post-weaning ADG, final BW, and higher serum glucose, BHB and insulin concentrations compared to animals in the SP-PLF group.

In contrast to PLF, starter intake during the pre-weaning period was greatly reduced by SBO. Moreover, an interaction between FPS × lipid supplement indicated that a long FPS, which was rather beneficial with PLF, enforced the negative effects of SBO on pre-weaning starter intake, resulting in a dramatic reduction in starter feed intake in group LP-SBO compared to LP-PLF by 270 g/d. When acknowledging that this period is critical for rumen development in young calves, it is understandable that performance parameter like ADG and withers height were reduced later in the post-weaning period in which animals of LP-SBO additionally showed the lowest value for feed conversion.

Soybean oil is rich in unsaturated FA in contrast to PLF that contains high level of saturated FA [12, 23, 38]. Unsaturated FAs were previously shown to reduce digestibility of NDF in dairy calves, which was exacerbated upon forage inclusion in starter diets [11]. This partly conforms with the results of the present study where a lower digestibility was indicated for OM and NDF selectively in calves receiving the SBO diet; however, only when FPS was long. This lower digestibility of nutrients is one likely reason that explains concurrent suppression of feed intake. Apart from calves [23], a feed intake depression due to unsaturated FAs is well documented in mature ruminants [39, 40]. Toxic compounds have been identified as a cause by which certain supplemental lipids can reduce NDF digestibility in ruminants [41]. It is perceivable that this phenomenon is aggravated in young dairy calves that have not fully established fiber-digesting bacteria in their rumen. The role of protozoa in fiber digestion can also be negatively affected by lipid supplementation in ruminants [42]. Collectively, the results support that LP may be more favorable than SP from the ruminal fermentation perspectives [8, 36], even if moderate amounts of saturated fatty acids are included in the diet. However, it is highly recommendable to reduce FPS when calves shall receive starters supplemented with oil rich in unsaturated fatty acids to avoid strong depressing effects on fiber digestion and feed intake. It may be speculated that unsaturated fatty acids can have stronger coating effect on LP vs. SP due to the smaller surface area of LP, leading to stronger impairment of microbial attachment on LP vs. SP.

According to the lowest starter intake in LP-SBO, the lowest ADG (658 g/d) during the post-weaning period was found in calves receiving LP-SBO, coinciding with lowest withers girth at weaning (90.7 cm) and final measurement (96.1 cm). These results show that feeding starters containing LP alfalfa hay with SBO supplementation provide insufficient nutrients for acquiring gain in young dairy calves. However, calves receiving alfalfa hay in the form of LP size had greater barrel size which may be related to greater gut fill rather than being an indicator of improved growth rate. Previous studies also stated that feeding forage to young dairy calves may confound results on growth performance due to increased fill of the gastrointestinal tract rather than a true effect on growth [8, 43].

Our result clarified that feeding SBO along with LP provided the lowest digestibility of OM (66.8%). We did not measure ruminal fermentation in the current study, but because ruminal fermentation and short fatty acid production is strongly dependent on OM fermentation in the rumen [44], a lower ruminal fermentation rate can be postulated for the LP-SBO diet. This postulate takes into account both the lower starter (i.e., OM) intake and the lower OM digestibility. In addition, looser feces in calves of the two SBO-supplemented groups pre-weaning, with highest fecal scores in the group SP-SBO, indicated that lower digestibility of OM and NDF is additionally related to higher passage rate, which would coincide with reduced microbial activity [23].

Glucose and BHB concentrations in the blood of calves fed the LP-SBO diet were reduced, which fits the lower starter intake detected in this group. As a result of lower starter intake and lower digestibility, fewer nutrients can be provided for absorption in the gastrointestinal tract and, hence, glucose and BHB were reduced as energy level indicators in the LP-SBO group. Higher concentrations of BUN in both groups supplemented with SBO compared to PLF indicate that ammonia nitrogen concentration was higher in the rumen of calves supplemented with the unsaturated FA source, which would be in line with previous studies [44]. This indicates lower nitrogen utilization efficiency in SBO vs. PLF diets due to lower microbial activity in the rumen.

In agreement with the reduced serum glucose and BHB concentrations, the concentration of insulin was also reduced (6.63 IU/L) when calves were fed LP-SBO. Because insulin is a function of glucose concentration, the lower insulin concentration logically followed the lower glucose concentration. Lower insulin concentration, in turn, may contribute to lower growth in young ruminants because of its role in glucose and amino acid metabolism that is critical in the early stage of growth [29, 45]. By contrast, the highest pre-weaning and overall concentrations of glucose, BHB and insulin were observed in group LP-PLF. This supports the notion that it is not the inclusion of LP but clearly the combination of LP with SBO that decreases metabolic and endocrine growth signals. In agreement with this postulate, a recent study by Takemura et al. [2] indicated that forage inclusion in starter diets of dairy calves increased growth hormone concentration in their blood.

Further indications for the mechanisms behind impaired performance of LP-SBO calves were obtained from the estimation of MPS. Calves in the group LP-SBO had lowest urinary allantoin and total urinary PD excretion during pre-weaning and post-weaning, indicating lowest MPS. The MPS estimated based on urinary PD can be used as an indicator for microbial ruminal development in young calves [30]. The reduction of allantoin and urinary PD excretion detected in calves fed LP-SBO thus suggests lower microbial activity as a plausible cause for decreased OM and NDF digestibility. Of note, starter intake and, as such, the amount of OM provided to microbes, was also reduced in the pre-weaning period in group LP-SBO, thus withdrawing OM from ruminal microbes as a key factor to support microbial protein synthesis [46].

A last aim of the present study was to assess the interaction effect of FPS × lipid on oxidative status. The concentrations of MDA were lower in the blood of calves that received PLF compared to SBO diets. MDA has been considered as an important indicator for oxidative damage in pre-weaned dairy calves [47]. Calving leads to oxidative stress, which can increase the formation of reactive oxygen species and overwhelm the antioxidant systems of neonate calves [47, 48]. Our results suggest that regardless of forage particle size, supplementing starters with unsaturated FA sources can aggravate oxidative stress in young calves, especially during the pre-weaning period. Although limited documents are available on the effect of supplemental lipid on oxidative stress indicators in dairy calves [48], Tsai et al. [49] indicated that SBO rich in linoleic acid has some pro-inflammatory effect in young ruminants that can modulate intake and performance in pre-weaning calves. The ruminal metabolism of FA should also be considered with caution when lipid supplementation is provided to dairy cows [50, 51], calves [52] or lambs [14, 15]. Most of the negative responses to supplemental unsaturated FA had been attributed to its negative influence on ruminal fermentation and microbial activity in calves under 2 months of age [13, 46]; however, it seems that additional effects on energy metabolism and its hormonal regulation, as well as on oxidative stress and possibly inflammation, should receive additional attention in future studies.

## Conclusions

A strong interaction of dietary forage particle size × lipid supplement was found in young dairy calves. With a rumen-inert fatty acid source (palm fatty acids), we observed a measurable increase of average daily gain, serum glucose, beta-hydroxy butyrate and insulin levels pre-weaning, as well as increased neutral detergent fiber digestibility and final body weight, when increasing particle size (1.26 mm to 4.97 mm) in a starter diet containing 10% alfalfa hay (dry matter basis). This is compatible with the effect of forage particle size observed using forage-supplemented starters without lipid supplementation in previous studies. By sharp contrast, addition of soybean oil to the starter diet had a generally depressing action on several of the above mentioned readouts that became obvious or was aggravated when increasing forage particle size. Therefore, supplementation with 25 g/kg palm fatty acids can be recommended to increase the energy density of forage-containing starter diets for dairy calves and preference should be given to a long forage particle size. On the contrary, negative performance effects of soybean oil as lipid supplement are especially evident with a long forage particle size. If soybean oil is to be used in starter diets for young dairy calves, the particle size should be short to avoid or ameliorate depressions of intake, digestibility, ruminal microbial activity, and growth performance.

## Data Availability

The datasets used and/or analyzed in the current study are available from the authors on reasonable request.

## Abbreviations

ADG: Average daily gain
BHB: Beta-hydroxy butyrate
BW: Body weight
CP: Crude protein
DM: Dry matter
FA: Fatty acid
FE: Feed efficiency
FPS: Forage particle size
GPx: Glutathione peroxidase
LP: Long particles
MDA: Malondialdehyde
MPS: Microbial protein synthesis
NASEM: National Academies of Science, Engineering, and Medicine
NDF: Neutral detergent fiber
NRC: National research council
OM: Organic matter
PD: Purine derivatives
PLF: Palm fatty acids
SBO: Soybean oil
SP: Small particles
SOD: Superoxide dismutase

## References

[1] Ferguson HJ, Koh-Tan HHC, Johnston PEJ, Wallace RJ, Andonovic I, Michie C (2022). Light microscopic observations of the ruminal papillae of cattle on diets with divergent forage to cereal ratios. Animal.

[2] Takemura K, Hiroyuki S, Hitishi M, Yo-Han K, Shiro K (2019). Effects of forage feeding on rumen fermentation, plasma metabolites, and hormones in Holstein calves during pre- and postweaning periods. J Anim Sci.

[3] Diao Q, Zhang R, Fu T (2019). Reviews of strategies to promote rumen development in calves. Animals.

[4] Hosseini H, Mirzaei-Alamouti H, Vazirigohar M, Mahjoubi E, Rezamand P (2019). Effects of whole milk feeding rate and straw level of starter feed on performance, rumen fermentation, blood metabolites, structural growth, and feeding behavior of Holstein calves. Anim Feed Sci Technol.

[5] Sun D, Yin Y, Guo C, Liu L, Mao S, Zhu W (2021). Transcriptomic analysis reveals the molecular mechanisms of rumen wall morphological and functional development induced by different solid diet introduction in a lamb model. J Anim Sci Biotechnol.

[6] Ghaffari MH, Kertz AF (2021). Review: Effects of different forms of calf starters on feed intake and growth rate: A systematic review and Bayesian meta-analysis of studies from 1938 to 2021. J Appl Anim Sci.

[7] Molaei M, Kazemi-Bonchenari M, Mirzaei M, Esmaeili HR (2021). The physical form of starter (finely ground versus pelleted) and alfalfa hay (chopped versus pelleted) in Holstein dairy calves: effects on growth performance, feeding behaviour, ruminal fermentation, and urinary purine derivatives. Anim Feed Sci Technol.

[8] Mirzaei M, Khorvash M, Ghorbani GR, Kazemi-Bonchenrai M, Riasi A, Nabipour A (2015). Effects of supplementation level and particle size of alfalfa hay on growth characteristics and rumen development in dairy calves. J Anim Physio Anim Nutr.

[9] National Research Council. Nutrient requirements of dairy cattle. 7th revised edition. Washington: National Academy Press; 2001.

[10] National Academies of Science, Engineering, and Medicine. Nutrient Requirements of Dairy Cattle. 8th revised edition. Washington: National Academy of Sciences; 2021. 38386771

[11] Karimi A, Alijoo YA, Kazemi-Bonchenari M, Mirzaei M, Sadri H (2021). Soybean oil supplementation and alfalfa hay inclusion in starter feed of Holstein dairy calves: growth performance, digestibility, ruminal fermentation and urinary purine derivatives. Ital J Anim Sci.

[12] Panahiha P, Mirzaei-Alamouti H, Kazemi-Bonchenari M, Aschenbach JR (2022). Growth performance, nutrient digestibility, and ruminal fermentation of dairy calves fed starter diets with alfalfa hay versus corn silage. J Dairy Sci.

[13] Keshavarz V, Dehghan-Banadaky M, Ganjkhanlou M, Kazemi-Bonchenari M (2023). Effects of deeding wheat straw or beet pulp in starters supplemented with either soybean oil or palm fatty acids on growth performance and urinary purine derivatives in dairy calves. Anim Feed Sci Technol.

[14] Bahramkhani-Zaringoli L, Mirzaei-Alamouti H, Aschenbach JR, Vazirigohar M, Patra AK, Jafari-Anarkooli I (2022). Effects of oil supplements on growth performance, ruminal fermentation, nutrient digestibility, feeding behavior, blood metabolites, and ruminal morphology in lambs during transition from a low- to a high-grain diet. Animals.

[15] Mirzaei-Alamouti H, Abdollahi A, Rahimi H, Moradi S, Vazirigohar M, Aschenbach JR (2021). Effects of dietary oil sources (sunflower and fish) on fermentation characteristics, epithelial gene expression and microbial community in the rumen of lambs fed a high-concentrate diet. Arch Anim Nutr.

[16] Anderson JL, Schossow CR, Rodriguez-Hernandez K, Lawrence RD, Sensevirathne ND (2019). Fat metabolism and utilization in growing dairy calves and heifers: what do we know and what still needs to be researched?. J Anim Sci.

[17] Ikwuegbu OA, Sutton JD (1982). The effect of varying the amount of linseed oil supplementation on rumen metabolism in sheep. Br J Nut.

[18] Kononoff PJ, Heinrichs AH, Buckmaster DR (2003). Modification of the Penn State forage and total mixed ration particle separator and the effects of moisture content on its measurements. J Dairy Sci.

[19] ASAE. S424.1. Method of determining and expressing particle size of chopped forage materials by sieving. St. Joseph: American Society of Agricultural and Biological Engineers; 1996.

[20] AOAC. Official Methods of Analysis. 17th ed. Arlington: AOAC International; 2002.

[21] Van Soest PJ, Roberts JB, Lewis BA (1991). Methods of dietary fiber, neutral detergent fibre and non-starch polysaccharides in relation to animal nutrition. J Dairy Sci.

[22] Van Keulen J, Young BA (1977). Acid insoluble ash as a natural marker for digestibility studies. J Dairy Sci.

[23] Ghorbani H, Kazemi-Bonchenari M, HosseinYazdi M, Mahjoubi E (2020). Effects of various fat delivery methods in starter diet on growth performance, nutrients digestibility and blood metabolites of Holstein dairy calves. Anim Feed Sci Technol.

[24] Khan MA, Lee HJ, Lee WS, Kim HS, Kim SB, Park SB (2008). Starch source evaluation in calf starter: II. Ruminal parameters, rumen development, nutrient digestibilities, and nitrogen utilization in Holstein calves. J Dairy Sci.

[25] Paglia DE, Valentine WN (1967). Studies on the quantitative and qualitative characterization of erythrocyte glutathione peroxidase. J Lab Clin Med.

[26] McCord JM, Fridovich I (1969). Superoxide dismutase. An enzymic function for erythrocuprein (hemocuprein). J Biol Chem.

[27] Placer ZA, Cushman LL, Johnson BC (1966). Estimation of product of lipid peroxidation (malonyl dialdehyde) in biochemical systems. Analyt Biochem.

[28] Dennis TS, Suarez-Mena FX, Hill TM, Quigley JD, Schlotterbeck RL, Lascano GJ (2018). Short communication: Effect of replacing corn with beet pulp in a high concentrate diet fed to weaned Holstein calves on diet digestibility and growth. J Dairy Sci.

[29] Kazemi-Bonchenari M, Dehghan-Banadaky M, Fattahnia F, Saleh-Bahmanpour A, Jahani-Moghadam M, Mirzaei M (2020). Effects of linseed oil and rumen-undegradable protein: rumen-degradable protein ratio on performance of Holstein dairy calves. Br J Nut.

[30] Kazemi-Bonchenari M, Khanaki H, Jafari A, Eghbali M, Poorhamdollah M, Ghaffari MH (2022). Milk feeding level and starter protein content: Effects on growth performance, blood metabolites, and urinary purine derivatives of Holstein dairy calves. J Dairy Sci.

[31] Funaba M, Kagiyama K, Iriki T, Abe M (1997). Duodenal flow of microbial nitrogen estimated from urinary excretion of purine derivatives in calves after early weaning. J Anim Sci.

[32] Gonzalez-Ronquillo M, Balcells J, Guada JA, Vicente F (2003). Purine derivative excretion in dairy cows: endogenous excretion and the effect of exogenous nucleic acid supply. J Dairy Sci.

[33] Chen XB, Gomes MJ. Estimation of microbial protein supply to sheep and cattle based on urinary excretion of purine derivatives: An overview of technical details. International Feed Research Unit, Rowett Research Institute, Aberdeen, UK, Occasional Publication. 1992. https://www.researchgate.net/publication/265323654.

[34] Francisco A, Alves SP, Portugal PV, Pires VMR, Dentinho MT, Alfaia CM (2016). Effect of feeding lambs with a tanniferous shrub (rockrose) and a vegetable oil blend on fatty acid composition of meat lipids. Animal.

[35] Aragona KM, Suarez-Mena FX, Dennis TS, Quigley JD, Hu W, Hill TM (2020). Effect of starter form, starch concentration, and amount of forage fed on Holstein calf growth from 2 to 4 months of age. J Dairy Sci.

[36] Nemati M, Amanlou H, Khorvash M, Moshiri B, Mirzaei M, Khan MA (2015). Rumen fermentation, blood metabolites, and growth performance of calves during transition from liquid to solid feed: Effects of dietary level and particle size of alfalfa hay. J Dairy Sci.

[37] Omidi-Mirzaei H, Azarfar A, Kiani A, Mirzaei M, Ghaffari MH (2018). Interaction between the physical forms of starter and forage source on growth performance and blood metabolites of Holstein dairy calves. J Dairy Sci.

[38] Hill TM, Bateman IHGI, Aldrich JM, Quigley JD, Schlotterbeck RL (2015). Inclusion of tallow and soybean oil to calf starters fed to dairy calves from birth to four months of age on calf performance and digestion. J Dairy Sci.

[39] Drackley JK, Klusmeyer TH, Trusk AM, Clark JH (1992). Infusion of long-chain fatty acids varying in saturation and chain length into abomasum of lactating dairy cows. J Dairy Sci.

[40] Harvatine KJ, Allen MS (2006). Effects of fatty acid supplements on feed intake, and feeding and chewing behavior of lactating dairy cows. J Dairy Sci.

[41] Soliva CR, Meile L, Cieslak A, Kreuzer M, Machmüller A (2004). Rumen simulation technique study on the interaction of dietary lauric and myristic acid supplementation in suppressing ruminal methanogenesis. Br J Nutr.

[42] Newbold CJ, de la Fuente G, Belanche A, Ramos-Morales E, McEwan NR (2015). The role of ciliate protozoa in the rumen. Front Microbiol.

[43] Clark JH, Klusmeyer TH, Cameron MR (1992). Microbial protein synthesis and flows of nitrogen fractions to the duodenum of dairy cows. J Dairy Sci.

[44] Yousefinejad S, Fattahnia F, Kazemi-Bonchenari M, Khanaki H, Drackley JK, Ghaffari MH (2021). Soybean oil supplementation and starter protein content: Effects on growth performance, digestibility, ruminal fermentation, and urinary purine derivatives of Holstein dairy calves. J Dairy Sci.

[45] Wester TJ, Lobley GE, Birine LM, Lomax MA (2000). Insulin stimulates phenylalanine uptake across the hind limb in fed lambs. J Nutr.

[46] Makizadeh H, Kazemi-Bonchenari M, Mansoori-Yarahmadi H, Fakhraei J, Khanaki H, Drackley JK (2020). Corn-processing and crude protein content in calf starter: Effects on growth performance, ruminal fermentation, and blood metabolites. J Dairy Sci.

[47] Gaal T, Ribiczeyne-Szabo P, Stadler K, Jakus J, Reiczigel J, Kover P (2006). Free radicals, lipid peroxidation and the antioxidant system in the blood of cows and newborn calves around calving. Comp Biochem Physiol B Biochem Mol Biol.

[48] Liu W, Ta LTZ, Evans A, Gao S, Yu Z, Bu D (2021). Supplementation with sodium butyrate improves growth and antioxidant function in dairy calves before weaning. J Anim Sci Biotech.

[49] Tsai CY, Rezamand P, Loucks WI, Scholte CM, Doumit ME (2017). The effect of dietary fat on fatty acid composition, gene expression and vitamin status in pre-ruminant calves. Anim Feed Sci Technol.

[50] Vazirigohar M, Dehghan-Banadaky M, Rezayazdi K, Krizsan SJ, Nejati-Javaremi A, Shingfield KJ (2014). Fat source and dietary forage-to-concentrate ratio influences milk fatty-acid composition in lactating cows. Animal.

[51] Vazirigohar M, Dehghan-Banadaky M, Rezayazdi K, Nejati-Javaremi A, Mirzaei-Alamouti H, Patra AK (2018). Short communication: Effects of diets containing supplemental fats on ruminal fermentation and milk odd- and branched-chain fatty acids in dairy cows. J Dairy Sci.

[52] Kazemi-Bonchenari M, Mirzaei M, Jahani-Moghadam M, Soltani A, Mahjoubi E, Patton RA (2016). Interactions between levels of heat-treated soybean meal and prilled fat on growth, rumen fermentation, and blood metabolites of Holstein calves. J Anim Sci.

